# Benefits of Sustained Upregulated Unimolecular GLP-1 and CCK Receptor Signalling in Obesity-Diabetes

**DOI:** 10.3389/fendo.2021.674704

**Published:** 2021-05-14

**Authors:** Neil Tanday, Andrew English, Ryan A. Lafferty, Peter R. Flatt, Nigel Irwin

**Affiliations:** Diabetes Research Group, School of Biomedical Sciences, Ulster University, Coleraine, United Kingdom

**Keywords:** GLP-1, exendin-4, CCK-8, diabetes, obesity

## Abstract

Combined activation of GLP-1 and CCK1 receptors has potential to synergistically augment the appetite-suppressive and glucose homeostatic actions of the individual parent peptides. In the current study, pancreatic beta-cell benefits of combined GLP-1 and CCK1 receptor upregulation were established, before characterising bioactivity and antidiabetic efficacy of an acylated dual-acting GLP-1/CCK hybrid peptide, namely [Lys^12^Pal]Ex-4/CCK. Both exendin-4 and CCK exhibited (p<0.001) proliferative and anti-apoptotic effects in BRIN BD11 beta-cells. Proliferative benefits were significantly (p<0.01) augmented by combined peptide treatment when compared to either parent peptide alone. These effects were linked to increases (p<0.001) in GLUT2 and glucokinase beta-cell gene expression, with decreased (p<0.05-p<0.001) expression of NFκB and BAX. [Lys^12^Pal]Ex-4/CCK exhibited prominent insulinotropic actions *in vitro*, coupled with beneficial (p<0.001) satiety and glucose homeostatic effects in the mice, with bioactivity evident 24 h after administration. Following twice daily injection of [Lys^12^Pal]Ex-4/CCK for 28 days in diabetic high fat fed (HFF) mice with streptozotocin (STZ)-induced compromised beta-cells, there were clear reductions (p<0.05-p<0.001) in energy intake and body weight. Circulating glucose was returned to lean control concentrations, with associated increases (p<0.001) in plasma and pancreatic insulin levels. Glucose tolerance and insulin secretory responsiveness were significantly (p<0.05-p<0.001) improved by hybrid peptide therapy. In keeping with this, evaluation of pancreatic histology revealed restoration of normal islet alpha- to beta-cell ratios and reduction (p<0.01) in centralised islet glucagon staining. Improvements in pancreatic islet morphology were associated with increased (p<0.05) proliferation and reduced (p<0.001) apoptosis of beta-cells. Together, these data highlight the effectiveness of sustained dual GLP-1 and CCK1 receptor activation by [Lys^12^Pal]Ex-4/CCK for the treatment of obesity-related diabetes.

## Introduction

The increasing use of GLP-1 mimetics to treat diabetes and obesity highlights the therapeutic importance of this class of drugs (1). Indeed, weight loss recently reported using the GLP-1 mimetic semaglutide in humans with obesity is highly impressive (2). Nevertheless, the pronounced metabolic benefits of bariatric surgery, often resulting in remission of diabetes, unquestionably exceed positive effects of GLP-1 mimetics, highlighting the important interplay between gut-derived hormones to induce distinct and sustained benefits in obesity-diabetes (3). In full agreement, administration of GLP-1 mimetics in combination with related gut-derived hormones imparts metabolic advantages beyond that seen with GLP-1 alone (4, 5). Recent advances in peptide drug design have also witnessed generation of several unimolecular dual- or triple-acting hybrid peptides, that contain a GLP-1 backbone structure (6). For example, Tirzepatide, a synthetic dual-acting GIP and GLP-1 receptor agonist, demonstrates remarkable antidiabetic effectiveness in phase II clinical trials (7). With such successes in mind, a strong rational exists for generating unimolecular agents that mimic meal-induced gut hormone release by co-activating receptors for GLP-1 plus other hormones involved in conveying the metabolic and satiety signals initiated by feeding.

Cholecystokinin (CCK) is a hormone primarily secreted from gastrointestinal I-cells as well as neurons of the enteric and central nervous systems (8). The peptide circulates in various molecular forms, with the octapeptide CCK-8 being the smallest form to exhibit full biological activity (9). To impart its physiological actions, CCK binds and activates specific receptors, namely CCK1 and CCK2, that modulate various physiological processes including satiety, pancreatic endocrine, and exocrine function as well as gastric acid and bile secretion (8). In particular, CCK-based regulation of energy intake and pancreatic endocrine islet function make the hormone a potentially attractive therapeutic agent for obesity and diabetes (9). As such, CCK dose-dependently inhibits food intake consistently across species, from rodents (10) to non-human primates (11) and humans (12) mediated *via* CCK1 receptors (13). CCK also stimulates insulin secretion in rodents and humans (14, 15), with sustained administration of a long-acting CCK1 selective receptor agonist exerting notable metabolic benefits in rodent models of type 2 diabetes (16, 17). In agreement, transgenic mice with genetically-induced CCK ablation (18) or overexpression (19) exhibit impaired or improved beta-cell function, respectively. Moreover, CCK has recently been shown to exert important beta-cell proliferative and anti-apoptotic effects (20), which might counter beta-cell loss in diabetes.

When taken together, the biological action profile of CCK has strong parallels with those of GLP-1 (9). Both hormones initiate separate, but complementary, cell signalling pathways *via* phospholipase C or adenylate cyclase, respectively (21). At the level of the pancreatic beta-cell an important GLP-1/CCK intra-islet loop has also been described that helps protect against beta-cell apoptosis, with obvious benefits for diabetes (21). Thus, it is unsurprising that combined activation of CCK1 and GLP-1 receptors has been noted to induce numerous benefits on body weight reduction and blood glucose control (22–24). Preliminary studies also provide proof-of-principle evidence for this therapeutic approach, both in terms of combined administration of individual long-acting GLP-1 and CCK1 agonists (5, 25), as well as dual-acting hybrid peptides (26, 27). This is highly relevant for clinical translation since methods to augment the antidiabetic effectiveness of approved GLP-1 therapeutics are much sought after (28).

Two separate dual-acting GLP-1/CCK fusion peptides have been described to date, with both exerting remarkable benefits on body weight reduction and pancreatic islet morphology, resulting in improved metabolic control in obese high fat fed mice (26, 27). Although uncharacterised in terms of *in vivo* islet effects, the fusion peptide described by Hornigold and colleagues (27) appeared to have the most attractive biological action profile, with confirmed balance between GLP-1 and CCK1 receptor activation. Based on this knowledge, and to progress attractiveness of the GLP-1/CCK hybrid peptide approach, we employed acylation of this molecule to create a compound capable of sustained, simultaneous activation of GLP-1 and CCK1 receptors, namely [Lys^12^Pal]Ex-4/CCK, with a pharmacodynamic profile more suitable for clinical development. Thus, a C-16 fatty acid was conjugated to the free amino group of Lys^12^ in the GLP-1/CCK hybrid peptide *via* a glutamyl linker (**Table 1**).

**Table 1:** Amino acid sequence of [Lys^12^Pal]Ex-4/CCK.

Peptide	Structure
[Lys^12^Pal]Ex-4/CCK	H-G-E-G-T-F-T-S-D-L-S-K(N-ϵ-(γ-GLU(hexadecanoyl))-Q-M-E-E-E-A-V-R-L-F-I-E-W-L-K-N-[PEG4]-Nle-G-W-K-D-NmeF

Sequence of [Lys^12^Pal]Ex-4/CCK using one letter amino acid notation. The C-16 fatty acid residue in [Lys^12^Pal]Ex-4/CCK is attached to Lys^12^ using a gamma-glutamyl spacer. Polyethylene glycol 4 (PEG4) is used as a linker. Nle, norleucine; NMeF, N-methylphenylalanine.

Our primary objective was to examine the therapeutic efficacy and islet cell morphology after 28 days twice daily treatment with [Lys^12^Pal]Ex-4/CCK in high fat fed (HFF) mice with limited beta-cell compensation induced by low-dose STZ administration. Prior to this, bioactivity of [Lys^12^Pal]Ex-4/CCK was established in BRIN BD11 beta-cells, alongside assessment of acute and more prolonged *in vivo* effects on satiety and glucose homeostasis in mice. In addition, the positive interplay between GLP-1 and CCK receptor signalling on beta-cell growth and survival, with related impact on gene expression, was also studied. Ultimately, our data confirm that the profound beneficial pharmacological effects of combined activation GLP-1 and CCK receptor pathways in obesity-diabetes can be effectively harnessed within an appropriately designed single long-acting hybrid peptide.

## Methods

### Peptides

[Lys^12^Pal]Ex-4/CCK (**Table 1**) and related peptides were synthesised by Synpeptide (Shanghai, China) at 95% purity and confirmed in-house by high-performance liquid chromatography (HPLC) and MALDI-TOF, as previously conducted (4).

### *In Vitro* Effects of GLP-1 and CCK1 Receptor Activation on Beta-Cell Gene Expression, Proliferation, and Apoptosis

Initial studies examined the impact of upregulated GLP-1 and CCK1 receptor signalling on the expression of key genes involved in insulin secretion and cell survival in BRIN BD11 beta-cells (29). Messenger RNA (mRNA) was isolated from cells (150,000 cells) following 18 h incubation with exendin-4, CCK-8, or a combination of both peptides (all at 10^-6^ M) using the RNeasy mini kit (Qiagen, UK) following manufacturer’s protocols. Utilising SuperScript™ II Reverse Transcriptase (Invitrogen Life Technologies, Carlsbad, CA, USA), mRNA was converted to cDNA as described previously. Expression of genes associated with apoptosis (BAX and NFκB) and insulin secretion (Glut2 and Glucokinase) were determined by quantitative real-time PCR, normalised to internal house-keeping control GAPDH (30). Primer sequences for genes studied were as previously described from our laboratory (31). To assess beta-cell proliferative effects, BRIN-BD11 cells (150,000 cells per chamber slide) were incubated for 18 h with test peptides (10^-6^ M) before fixation in paraformaldehyde and staining for Ki-67 (Ab15580, AbCam). Likewise, to assess effects on protection against apoptosis, cells were cultured in cytokine mix (IL-1β 100U/ml, IFN-γ 20 U/ml, TNF-α 200 U/ml) alone or with test peptides for 18 h, prior to fixation and staining for TUNEL using an *in-situ* death detection kit (*in situ* cell death kit, Fluorescein; Roche Diagnostics). Both Ki-67 and TUNEL stained cells were imaged using a fluorescence microscope (Olympus system microscope, model BX51) fitted with DAPI (350 nm), FITC (488 nm) and TRITC (594 nm) filters and an Olympus XM10 camera system.

### *In Vitro* Effects of [Lys^12^Pal]Ex-4/CCK on Insulin Secretion

To examine effects of [Lys^12^Pal]Ex-4/CCK on insulin secretion, BRIN-BD11 cells were seeded at a density of 150,000 cells per well into 24-well plates and allowed to attach overnight. Following a 40-minute preincubation (1.1 mM glucose KRB buffer), cells were incubated with KRB buffer (16.7 mM glucose) with [Lys^12^Pal]Ex-4/CCK or exendin-4 (10^-6^ M). In a second experiment cells were incubated with classical modulators of insulin secretion in combination with [Lys^12^Pal]Ex-4/CCK or exendin-4 (each at 10^-6^ M). For all experiments, BRIN-BD11 cells were incubated under test conditions for a 20-minute period at 37°C, with the resulting supernatant collected and insulin content assessed by radioimmunoassay (32).

### Acute *In Vivo* Experiments

Acute *in vivo* studies were carried out using 10-week-old adult male C57BL/6 mice (Envigo Ltd, UK). Mice were single-housed and kept in a temperature-controlled environment (22 ± 2°C) under a 12-hour light/dark cycle, with *ad libitum* access to drinking water and maintenance diet (10% fat, 30% protein and 60% carbohydrate, percent of total energy 12.99 kJ/g; Trouw Nutrition, UK). All experiments were approved by Ulster University Animal Ethics Review Committee and conducted in accordance with the UK Animals (Scientific Procedures) Act 1986. To assess effects of [Lys^12^Pal]Ex-4/CCK on food intake in overnight (18 hour) fasted mice, mice (n=8) were administered with an intraperitoneal (i.p.) injection of saline vehicle (0.9% [w/v] NaCl) or [Lys^12^Pal]Ex-4/CCK (25 nmol/kg bw) and cumulative food consumption recorded at regular intervals over a 180 min observation period. Acute effects of [Lys^12^Pal]Ex-4/CCK on glucose homeostasis were also assessed in overnight fasted mice, following an i.p. injection of glucose alone (18 mmol/kg bw) or in combination with [Lys^12^Pal]Ex-4/CCK (25 nmol/kg bw). More persistent effects on feeding and glucose tolerance were examined as above, but with peptide injected 4, 8, 12 or 24 h prior to feeding or administration of glucose, as appropriate. In a separate series of experiments, the impact of established GLP-1 and CCK1 receptor antagonists (both at 25 nmol/kg bw), namely exendin(9-39) and SR27897 (Tocris Bioscience, Bristol, UK) respectively, on [Lys^12^Pal]Ex-4/CCK induced (25 nmol/kg bw) satiety was examined.

### Chronic *In Vivo* Experiments

Adult male NIH Swiss mice (12-week-old), were maintained for 3 weeks on high fat diet (45% fat, 20% protein, 35% carbohydrate; percent of total energy 26.15 kJ/g; Dietex International Ltd., Witham, UK). After this period, they were administered three once weekly i.p. injections of streptozotocin (4-hour fast, 50 mg/kg bw, dissolved in sodium citrate buffer, pH 4.5) on weeks 3, 4 and 5. Starting on week 6, diabetic mice (non-fasting glycaemia >11.1 mmol/l) were grouped (n=8) and received twice-daily intraperitoneal injections (08:00 and 20:00) of saline vehicle (0.9% [w/v] NaCl) or [Lys^12^Pal]Ex-4/CCK (25 nmol/kg bw) for 28 days. Mice were continued on high fat diet throughout the experiment. At regular intervals, cumulative energy intake and body weight were assessed, with circulating glucose, insulin and glucagon measured on day 28. At the end of the treatment period, glucose tolerance (18 mmol/kg bw; i.p.; 18-h fasted) and insulin sensitivity (25 U/kg bovine insulin; i.p.; non-fasted) tests were conducted. Terminal analyses involved dissection of pancreatic tissue, which was processed for quantification of hormone content or pancreatic islet morphology, following acid ethanol protein extraction or fixation in 4% PFA, respectively (33).

### Biochemical Analyses

Blood samples were obtained from conscious mice *via* the cut tip on the tail vein and blood glucose immediately measured using an Ascencia Contour blood glucose meter (Bayer Healthcare Newbury, UK). Blood was collected in chilled heparin/fluoride coated micro-centrifuge tubes (Sarstedt, Numbrecht, Germany) and centrifuged for 15 minutes at 12,000 rpm using a Beckman microcentrifuge (Beckman Instruments, Galway, Ireland) to separate plasma. Insulin and glucagon were then measured by radioimmunoassay (32) or commercially available ELISA (EZGLU-30K, Merck Millipore, Burlington, Massachusetts), respectively.

### Immunohistochemistry

Immunohistochemistry was used to examine islet morphology by staining for insulin (1:400; ab6995, AbCam) or glucagon (1:1000; ab92517, AbCam). Image analysis was carried out using Cell^F^ image analysis software (Olympus Soft Imaging Solutions, GmbH, Münster, Germany). To assess islet morphology, areas of insulin and glucagon positive staining were quantified using a “closed polygon” and expressed as islet/beta-/alpha- cell areas in µm^2^, as described previously (34). To assess beta-cell proliferation and apoptosis, co-staining of insulin with Ki-67 (1:400; ab15580, AbCam) or TUNEL (*In situ* cell death kit, Fluorescein; Roche Diagnostics) was conducted. For quantification, the number of insulin-positive cells co-expressing Ki-67 or TUNEL respectively were counted using ImageJ software, with >80 islets analysed per treatment group. In all cases, following incubation with primary antibodies, the following secondary antibodies Alexa Fluor594 goat anti-mouse IgG and Alexa Fluor488 goat anti-rabbit, were used as appropriate (1:400; ThermoFisher Scientific). Slides underwent a final incubation with DAPI before being mounted for imaging using a fluorescence microscope (Olympus system microscope, model BX51) fitted with DAPI (350 nm), FITC (488 nm) and TRITC (594 nm) filters and an Olympus XM10 camera system.

### Statistical Analyses

Statistical tests were conducted using GraphPad PRISM software (Version 5.0). Values are expressed as mean ± SEM. Comparative analyses between groups were performed using a one-way ANOVA with Bonferroni’s *post hoc* test, a two-way ANOVA with Bonferroni’s *post hoc* test or Student’s unpaired t-test, as appropriate. Differences were deemed significant if p<0.05.

## Results

### Effects of Individual and Combined Upregulation of GLP-1 and CCK1 Receptors on Beta-Cell Proliferation and Apoptosis, as well as Related Gene Expression

**Figure 1:**
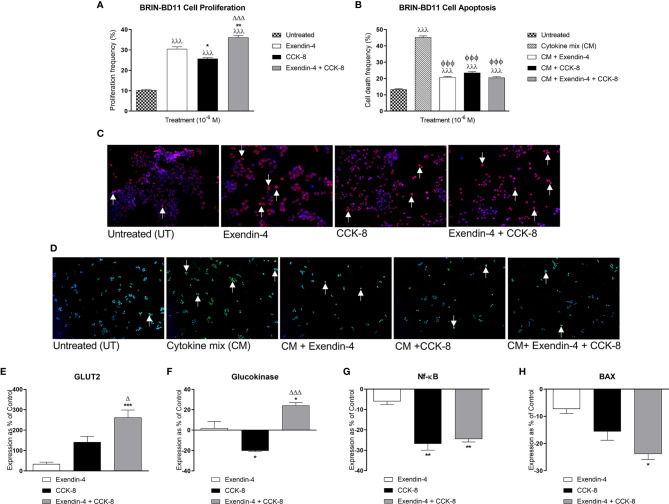
Effects of exendin-4, CCK-8, and a combination of both peptides on BRIN-BD11 proliferation, apoptosis, and expression of key genes. **(A, B)** To assess effects on BRIN BD11 beta-cell proliferation and apoptosis, cells were incubated with test peptides (10^-6^ M) alone, or together with cytokine mix (CM; IL-1β 100U/ml, IFN-γ 20 U/ml, TNF-α 200 U/ml) where specified, for 18 h prior to staining for **(A)** Ki-67 or **(B)** TUNEL. Representative images show cells stained for **(C)** Ki-67 (red) and **(D)** TUNEL (green) with DAPI (blue), with arrows indicating positively stained cells. **(E–H)** qPCR analysis of Glut2, glucokinase, NFκB and Bax mRNA expression following 18 h incubation with test peptides (10^-6^ M) in BRIN BD11 beta-cells. All gene expression data was normalised to GAPDH housekeeping control. Values are mean ± SEM (n=4). ^λλλ^p < 0.001 compared to untreated controls. ^ΦΦΦ^p < 0.001 compared to cytokine mix. *p < 0.05, **p < 0.01, ***p < 0.001 compared to exendin-4 alone. ^Δ^p < 0.05 and ^ΔΔΔ^p < 0.001 compared to CCK-8 alone.

Exendin-4 and CCK-8 increased (p<0.001) BRIN-BD11 beta-cell proliferation (**Figure 1A**). More interestingly, co-incubation of exendin-4 with CCK-8 induced greater beta-cell proliferative effects when compared to exendin-4 (p<0.01) or CCK-8 (p<0.001) alone (**Figure 1A**). Likewise, both peptides reduced (p<0.001) cytokine-induced BRIN-BD11 cell apoptosis, with combined exendin-4 and CCK-8 treatment evoking comparable benefits to the parent peptides alone (**Figure 1B**). Representative images of BRIN-BD11 cells stained for Ki-67 or TUNEL under each incubation condition are presented in **Figures 1C, D**, respectively. Exendin-4 and CCK-8 had no significant effect of Glut2 expression following an 18 h incubation in BRIN BD11 beta-cells, but combined peptide treatment significantly increased Glut2 mRNA levels when compared to exendin-4 (p<0.001) or CCK-8 (p<0.05) alone (**Figure 1E**). Whilst CCK-8 decreased (p<0.05) glucokinase expression, combined treatment with exendin-4 increased glucokinase gene expression compared to exendin-4 (p<0.05) or CCK-8 (p<0.001) alone (**Figure 1F**). Both CCK-8 alone, and in combination with exendin-4, decreased (p<0.01) NFκB expression (**Figure 1G**). Neither exendin-4 nor CCK-8 alone significantly affected Bax expression in BRIN BD11 cells, but combined peptide treatment resulted in a significant (p<0.05) decrease in Bax mRNA levels (**Figure 1H**).

### *In Vitro* Insulinotropic Actions of [Lys^12^Pal]Ex-4/CCK

**Figure 2:**
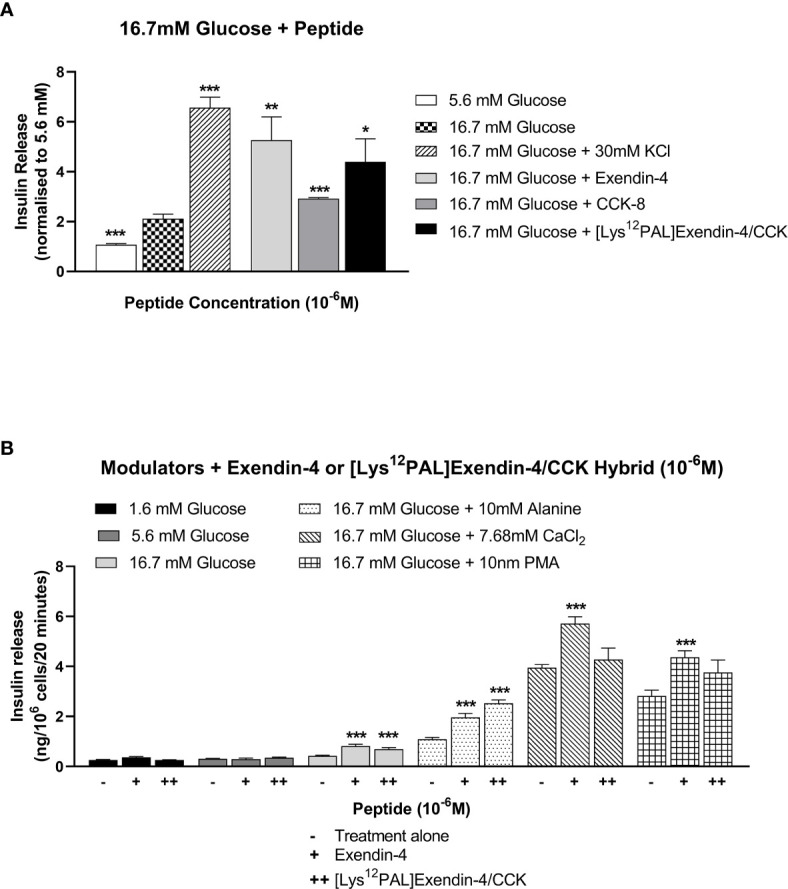
Effects of exendin-4, CCK-8 or [Lys^12^Pal]Ex-4/CCK on insulin release from rodent BRIN-BD11 cells. **(A)** Cells were incubated (20 min) with 16.7 mM glucose alone and with exendin-4, CCK-8 or [Lys^12^Pal]Ex-4/CCK (10^-6^ M) and insulin secretion assessed by RIA. **(B)** Effects of exendin-4 or [Lys^12^Pal]Ex-4/CCK (10^-6^ M) on the secretory action of established modulators of insulin secretion in BRIN BD11 cells. Values are mean ± SEM (n=8). *p < 0.05, **p < 0.01 and ***p < 0.001 compared with **(A)** 16.7 mM glucose alone or **(B)** insulinotropic agent with peptide.

Similar to exendin-4, and to a lesser extent CCK-8, [Lys^12^Pal]Ex-4/CCK augmented (p<0.05 – p<0.01) 16.7 mM glucose-induced insulin release from BRIN-BD11 cells at a concentration of 10^-6^ M (**Figure 2A**). [Lys^12^Pal]Ex-4/CCK and exendin-4 also exerted relatively similar insulin secretory potentiating actions (p<0.001) on the stimulatory effects of key modulators of insulin release, namely 16.7 mM glucose, 10 mM alanine, 7.68 mM Ca^2+^ and 10 nM PMA (**Figure 2B**).

### Acute Effects of [Lys^12^Pal]Ex-4/CCK on Food Intake and Glucose Tolerance

**Figure 3:**
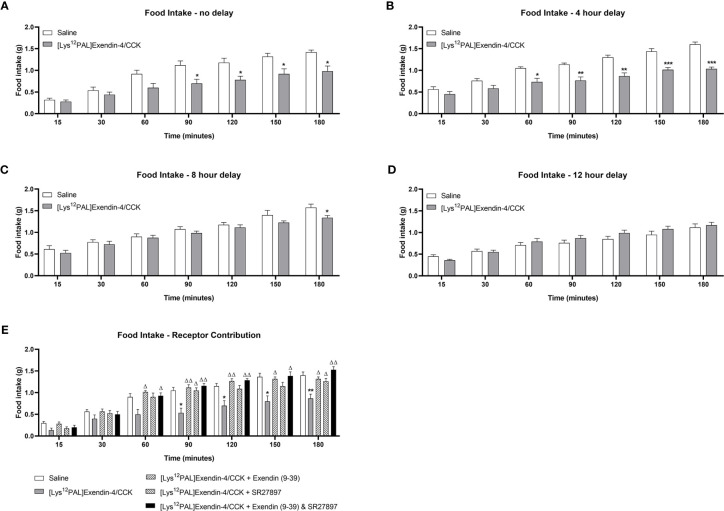
Effects of [Lys^12^Pal]Ex-4/CCK on food intake in mice. [Lys^12^Pal]Ex-4/CCK (25 nmol/kg bw, i.p.) was administered **(A)** 0, **(B)** 4, **(C)** 8 or **(D)** 12 h prior to assessment of food intake in overnight (16 h) fasted C57BL/6 mice. **(E)** [Lys^12^Pal]Ex-4/CCK (25 nmol/kg bw, i.p.) was administered alone or together with either exendin(9-39) or SR27897 (both at 25 nmol/kg bw) as well as in combination with both exendin(9-39) and SR SR27897. Food intake was recorded at regular intervals over a 3-hour observation period. Values are mean ± SEM for 8 mice. *p < 0.05, **p < 0.01 and ***p < 0.001 compared with saline-treated control. ^Δ^p < 0.05 and ^ΔΔ^p < 0.01 compared with [Lys^12^PAL]Ex-4/CCK.

**Figure 4:**
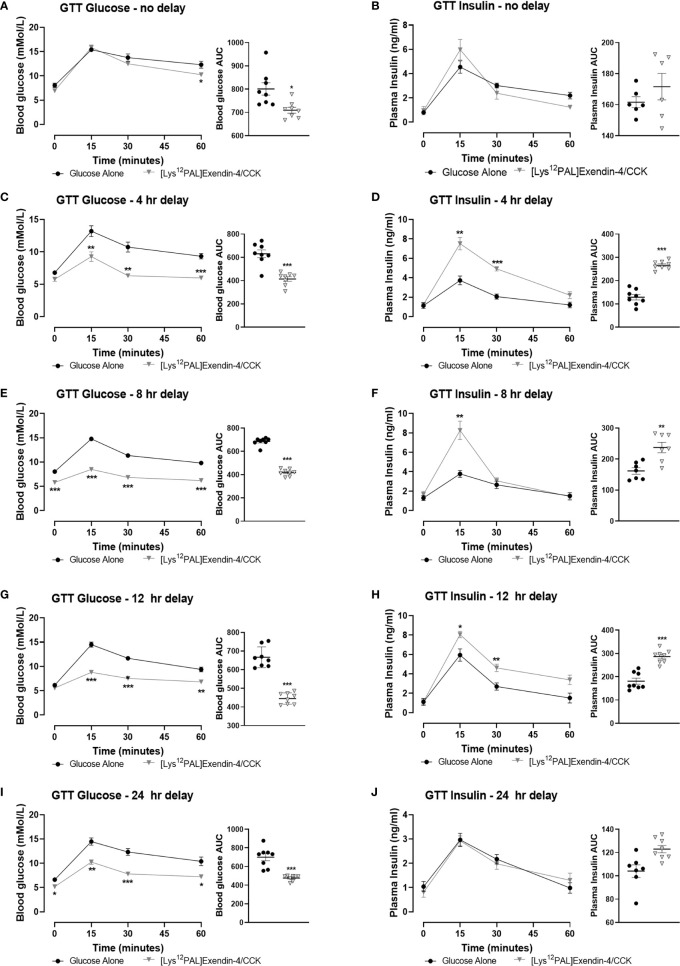
Effects of [Lys^12^Pal]Ex-4/CCK on glucose tolerance and insulin secretion in C57BL/6 mice. Blood glucose and plasma insulin concentrations were assessed following administration of [Lys^12^Pal]Ex-4/CCK (25 nmol/kg bw, i.p.) in combination **(A, B)** with glucose (18 mmol/kg bw, i.p.) or **(C, D)** 4 h, **(E, F)** 8 h, **(G, H)** 12 h or **(I, J)** 24 h prior to glucose administration. 0-60 min glucose AUC values are also shown. Values are mean ± SEM for 8 mice. *p < 0.05, **p < 0.01 and ***p < 0.001 compared with glucose alone control.

When injected i.p. in overnight fasted mice, [Lys^12^Pal]Ex-4/CCK reduced (p<0.05) food intake at all observation points from 90 min onwards (**Figure 3A**). Moreover, [Lys^12^Pal]Ex-4/CCK displayed persistent satiety effects in mice when administered 4 (p<0.001) or (p<0.05) 8 h prior to feeding (**Figures 3B, C**). When [Lys^12^Pal]Ex-4/CCK was delivered 12 h prior to feeding, the ability to inhibit food intake were no longer apparent (**Figure 3D**). Studies with GLP-1 and CCK1 receptor antagonists suggested that the satiating actions of [Lys^12^Pal]Ex-4/CCK were dependent on both GLP-1 and CCK1 receptor signalling, with perhaps marginally more reliance on GLP-1 receptor signalling in this regard (**Figure 3E**). When injected co-jointly with glucose [Lys^12^Pal]Ex-4/CCK reduced (p<0.05) individual and AUC glucose values, but this was not associated with demonstrably increased plasma insulin concentrations (**Figures 4A, B**). However, administration of [Lys^12^Pal]Ex-4/CCK either 4, 8 or 12 h prior to the glucose challenge resulted in robust (p<0.01–p<0.001) glucose-lowering and insulin secretory actions (**Figures 4C–H**). Interestingly, [Lys^12^Pal]Ex-4/CCK still possessed significant (p<0.001) glucose homeostatic effects when administered 24 h prior to glucose (**Figure 4I**), although no demonstrable change of glucose-induced insulin release was observed at this time (**Figures 4I–J**). This contrasts with the comparatively transient effects of native exendin-4 and CCK-8 as reported previously (16, 35).

### Effects of [Lys^12^Pal]Ex-4/CCK on Body Weight, Food Intake as well as Circulating Glucose, Insulin, and Glucagon

**Figure 5:**
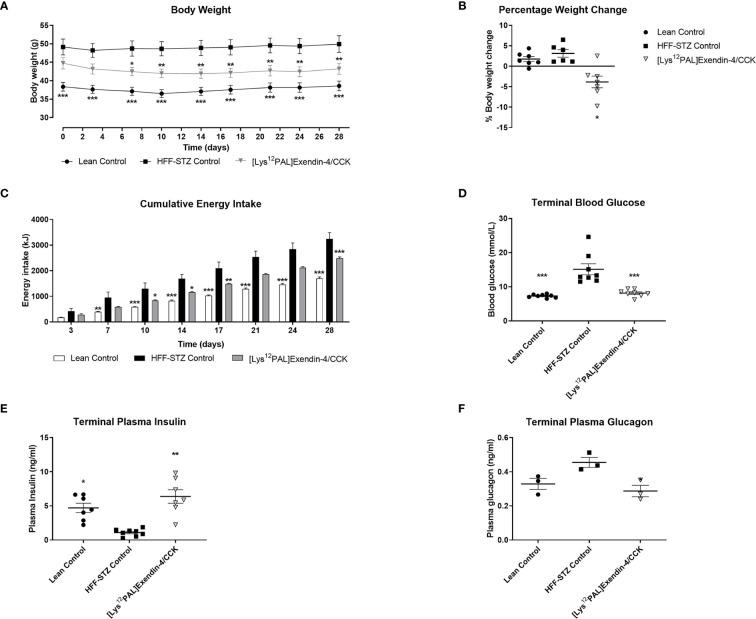
Effects of [Lys^12^Pal]Ex-4/CCK on **(A)** body weight, **(B)** body weight change, **(C)** calorie consumption, **(D)** terminal non-fasting blood glucose as well as terminal non-fasting plasma **(E)** insulin and **(F)** glucagon in HFF-STZ mice. **(A–C)** Parameters were measured at regular intervals over 28 days treatment with twice-daily [Lys^12^Pal]Ex-4/CCK (25 nmol/kg bw, i.p) in HFF-STZ mice. **(D–F)** Parameters were measure on day 28, with plasma insulin and glucagon were measured by RIA or ELISA, respectively. Values are mean ± SEM for 8 mice. *p < 0.05, **p < 0.01 and ***p < 0.001 compared with HFF-STZ diabetic control.

Twice daily treatment with [Lys^12^Pal]Ex-4/CCK in HFF mice with STZ-induced compromised beta-cells, namely HFF-STZ mice, resulted in significant (p<0.05) weight loss on day 7, that persisted until the end of the study (**Figure 5A**). Interestingly, there was a small decline of body weight in all mice on day 3, likely as a result of treatment regimen acclimatisation, with body weights of saline treated mice then stabilising compared with a continued gradual reduction of weight in mice receiving [Lys^12^Pal]Ex-4/CCK (**Figure 5A**). Indeed, when body weight change over the 28 days was analysed, only [Lys^12^Pal]Ex-4/CCK treated HFF-STZ mice presented with a reduction (p<0.05) in body weight gain (**Figure 5B**). [Lys^12^Pal]Ex-4/CCK induced body weight reductions were associated with consistent decreases (p<0.05-p<0.001) in energy intake (**Figure 5C**). On day 28, circulating glucose in [Lys^12^Pal]Ex-4/CCK HFF-STZ mice was similar to lean controls, and significantly (p<0.001) reduced when compared to HFF-STZ control mice (**Figure 5D**). Corresponding plasma insulin levels were decreased in HFF-STZ control mice, and elevated (p<0.01) to levels above lean controls by [Lys^12^Pal]Ex-4/CCK intervention (**Figure 5E**). Interestingly, plasma glucagon was not different between lean and diabetic control mice on day 28, but [Lys^12^Pal]Ex-4/CCK decreased (p<0.05) circulating glucagon when compared to HFF-STZ control mice (**Figure 5F**).

### Effects of [Lys^12^Pal]Ex-4/CCK on Glucose Tolerance, Insulin Sensitivity, and Pancreatic Insulin and Glucagon Content

**Figure 6:**
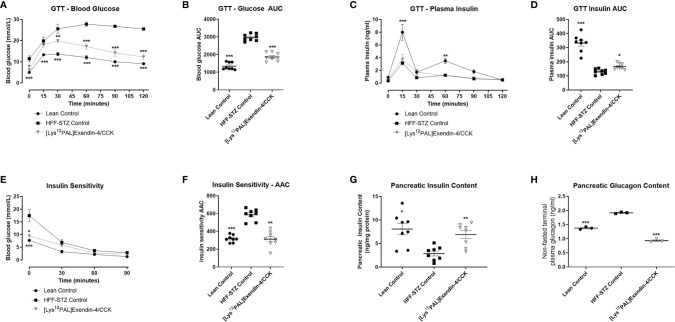
Effects of [Lys^12^Pal]Ex-4/CCK on **(A, B)** glucose tolerance, **(C, D)** insulin secretion, **(E, F)** as well as pancreatic **(G)** insulin and **(H)** glucagon content in HFF-STZ mice. Parameters were measured following 28 days twice-daily treatment with [Lys^12^Pal]Ex-4/CCK (25 nmol/kg bw, i.p). **(A, B)** Blood glucose and **(C, D)** plasma insulin was measured prior to and after administration of glucose alone (18 mmol/kg, i.p) at t = 0 min. **(E, F)** Blood glucose was measured after administration of insulin (25 U/kg bw, i.p) at t = 0 min. **(G, H)** Pancreatic insulin and glucagon content were measured by RIA or ELISA, respectively, following pancreatic protein extraction. Values are mean ± SEM for 8 mice. *p < 0.05, **p < 0.01 and ***p < 0.001 compared with HFF-STZ diabetic control.

Following a glucose challenge on day 28, [Lys^12^Pal]Ex-4/CCK mice presented with substantially (p<0.001) improved glucose homeostasis (**Figures 6A, B**), that was associated with a significant (p<0.05) augmentation of glucose-induced insulin release (**Figures 6C, D**), when compared to HFF control mice with STZ-induced compromised beta-cells. However, glucose tolerance and insulin secretion were still impaired (p<0.001) in [Lys^12^Pal]Ex-4/CCK HFF-STZ mice when compared to lean controls (**Figures 6A, D**). On first inspection, peripheral insulin sensitivity appeared to be largely unaffected by [Lys^12^Pal]Ex-4/CCK treatment, but when considered as glycaemic response with AAC analysis, a clear improvement (p<0.01) was evident (**Figures 6E, F**). This improvement appeared to be partly driven by clear reductions of non-fasting glucose levels evoked by 28 days treatment with [Lys^12^Pal]Ex-4/CCK in HFF/STZ mice. Pancreatic insulin content was elevated (p<0.01), and glucagon content decreased (p<0.001), in [Lys^12^Pal]Ex-4/CCK treated mice when compared to HFF-STZ controls, with values not significantly different from lean control mice (**Figures 6G, H**).

### Effects of [Lys^12^Pal]Ex-4/CCK on Pancreatic Islet Morphology as well as Beta-Cell Proliferation and Apoptosis

**Figure 7:**
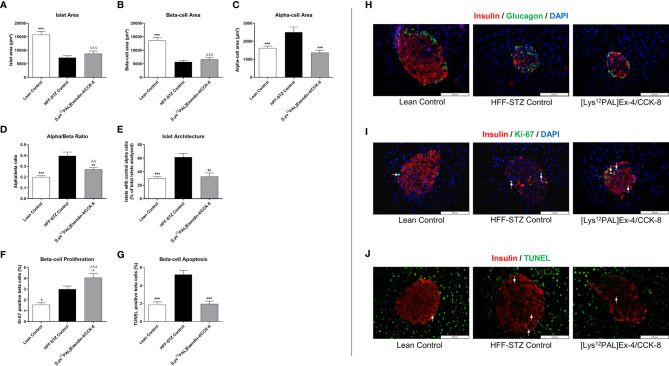
Effects of [Lys^12^Pal]Ex-4/CCK on pancreatic islet morphology as well as beta-cell proliferation and apoptosis in HFF-STZ diabetic mice. **(A)** Islet-, **(B)** beta- and **(C)** alpha-cell areas, **(D)** alpha:beta ratio, **(E)** percentage of glucagon positive centrally stained cells as well as beta-cell **(F)** proliferation and **(G)** apoptosis were measured following 28 days twice-daily treatment with [Lys^12^Pal]Ex-4/CCK (25 nmol/kg bw, i.p). Islet morphology was assessed using Cell^F^ image analysis software, with beta-cell proliferation and apoptosis measured Ki-67 or TUNEL staining, respectively. **(H-J)** Representative islet images (x40 magnification) show **(H)** insulin (red) and glucagon (green), **(I)** insulin (red) and Ki-67 (green) or **(J)** insulin (red) and TUNEL (green) from each treatment group. Values are mean ± SEM for 8 mice. *p < 0.05, **p < 0.01 and ***p < 0.001 compared with HFF-STZ diabetic control. ^ΔΔ^p < 0.01 and ^ΔΔΔ^p < 0.001 compared with lean control.

HFF-STZ mice had reduced (p<0.001) islet and beta-cell areas, that were not significantly affected by [Lys^12^Pal]Ex-4/CCK treatment (**Figures 7A, B**). However, elevations (p<0.001) of alpha-cell area in HFF-STZ mice were fully reversed by [Lys^12^Pal]Ex-4/CCK treatment (**Figure 7C**), which was associated with a trend towards normalisation of alpha:beta ratio in these mice (**Figure 7D**). Moreover, the characteristic appearance of increased glucagon staining within the centre of islets in HFF-STZ mice was reversed by [Lys^12^Pal]Ex-4/CCK treatment (**Figure 7E**). In addition, beta-cell proliferation was increased (p<0.05) and beta-cell apoptosis decreased (p<0.001), respectively, by [Lys^12^Pal]Ex-4/CCK treatment compared to HFF/STZ mice (**Figures 7F, G**), with beta-cell proliferation also increased (p<0.001) when compared to lean control mice (**Figure 7F**). Representative images of islets stained for insulin and glucagon as well as insulin and Ki-67 or insulin and TUNEL are presented in **Figures 7H–J**.

## Discussion

Additive, and frequently synergistic, benefits on satiety and pancreatic endocrine function by combined GLP-1 and CCK1 receptor activation have been documented previously (23, 36). In the current study, we have exploited this in terms of realising a new avenue for the treatment of T2DM. First, we examined the potential benefits of upregulated GLP-1 and CCK1 receptor activation on pancreatic beta-cells, given the recent observations that CCK may be less influential in terms of augmenting GLP-1 induced beta-cell insulin secretion (37). This is an important aspect to understand because GLP-1 and CCK are not only synthesised in the intestine but also released locally by islet cells to exert autocrine or paracrine actions (19, 20).

Independent benefits on beta-cell growth and survival following upregulation of GLP-1 and CCK receptor activation were verified (18, 34). Although additive effects were not apparent in terms of protection against apoptosis, there were clear benefits of combined receptor upregulation on beta-cell proliferation. Interestingly, interactions between islet-derived GLP-1 and CCK have been suggested to be important for beta-cell survival, rather than growth (21). Our contrasting observations may be linked to the experimental system employed, as well as differences in duration and extent of receptor activation. However, proliferative benefits of GLP-1 and CCK in the current setting are fully supported by changes in gene expression. As such, beta-cell regeneration is believed to be controlled by glucose metabolism and metabolic demand (38), with combination GLP-1 and CCK-8 treatment resulting in distinct increases of glucose sensor glucokinase gene expression (39). Combined treatment also resulted in a reduction in pro-apoptotic NFκB and BAX gene expression in line with known downstream actions of CCK (40). When viewed in conjunction with previous observations (5, 25), these data provide a compelling case to further explore the therapeutic potential of GLP-1/CCK dual-acting hybrid peptides (26, 27).

To this end, [Lys^12^Pal]Ex-4/CCK was engineered as a long-acting GLP-1 and CCK1 receptor agonist (**Table 1**), based on earlier positive observations of pronounced bioactivity of a GLP-1/CCK-8 fusion peptide and the pharmacodynamic benefits of peptide acylation (27, 41). As such, [Lys^12^Pal]Ex-4/CCK combines the key complementary glucose-lowering and satiety actions of exendin-4 and CCK-8, possessing an exendin-4 like N-terminus fused to a C-terminal CCK1 receptor agonist through use of a polyether linking compound (**Table 1**). Consistent with previous studies, activation of GLP-1 and CCK1 receptors by [Lys^12^Pal]Ex-4/CCK stimulated insulin secretion from BRIN BD11 cells (5, 26). Given that [Lys^12^Pal]Ex-4/CCK is acylated at Lys^12^ to promote albumin binding and prolong half-life, an intervention which often perturbs bioactivity (41), equipotency with exendin-4 is encouraging. To further probe insulinotropic effects, [Lys^12^Pal]Ex-4/CCK was co-incubated with known modulators of beta-cell function and shown to potentiate the insulin releasing actions of glucose, alanine, elevated Ca^2+^ or the phorbol ester PMA. Further to these *in vitro* effects, acute administration of [Lys^12^Pal]Ex-4/CCK to mice inhibited feeding activity, improved glucose tolerance, and potentiated the plasma insulin response to glucose. Such actions were protracted, with some effects even persisting when the peptide was administered up to 24 hours previously, contrasting with actions of native exendin-4 and CCK-8 which are relatively short-lived under similar experimental conditions (16, 35). Experiments employing pharmacological GLP-1 and CCK1 receptor antagonists perhaps suggested a very slight bias towards GLP-1 receptor signalling in mediating the biological actions of [Lys^12^Pal]Ex-4/CCK, in agreement with previous reports (27), but genuine prudence is required in terms of this data interpretation. Thus, the recognised synergism between GLP-1 and CCK receptor pathways (21–23) is not quantifiable when employing knockout of individual receptor pathways. Although informative, these antagonist studies are unable to provide a complete picture of the undoubted importance of synergy between GLP-1 and CCK1 receptor pathways for [Lys^12^Pal]Ex-4/CCK mediated benefits (26, 27).

Encouraged by these observations, we embarked on a longer-term *in vivo* study in mice that were administered low dose injections of STZ daily over 14 days to compromise beta-cell function in association with *ad libitum* access to high fat diet to induce insulin resistance and beta-cell stress (33). The resulting phenotype was characterised by failure of classical islet hypertrophy and beta-cell compensation in HFF-STZ mice, culminating in severe hyperglycaemia with blood glucose >12 mmol/l. Twice daily injection of these mice with [Lys^12^Pal]Ex-4/CCK for 28 days led to significant reductions in energy intake, body weight and blood glucose levels, in keeping with upregulated GLP-1 and CCK1 receptor activity (9). Importantly, these beneficial effects were apparent early in the treatment regimen and persisted throughout, with no indication of desensitisation (42). This highlights the long-acting nature and efficacy of [Lys^12^Pal]Ex-4/CCK. Development of a specific assay to measure [Lys^12^Pal]Ex-4/CCK in plasma, as well as tissue distribution, would be useful to provide more precise details of *in vivo* bioavailability.

Improvements in glucose tolerance in HFF mice with STZ-induced compromised beta-cells, although likely to be partially dependent on weight loss (43), were associated with significantly increased glucose-induced insulin concentrations. Indeed, circulating and pancreatic insulin were substantially increased in [Lys^12^Pal]Ex-4/CCK mice at the end of the 28-day study. It would also have been useful to examine metabolic effects following an oral nutrient challenge, but unfortunately that was not possible in the current study. Interestingly, the characteristic insulin resistance evoked by sustained high fat feeding (44) did not appear to be appreciably improved by [Lys^12^Pal]Ex-4/CCK intervention. Evidence for a small but significant enhancement of AAC glucose was noted, but this modest effect could be related to the fact that blood glucose levels in [Lys^12^Pal]Ex-4/CCK treated mice were well below 5 mmol/l during the experiment. This presumably resulted in activation of adaptive responses to counter the hypoglycaemia induced by bolus injection of exogenous insulin. In contrast to the small magnitude of this effect, [Lys^12^Pal]Ex-4/CCK evoked particularly prominent improvements in islet architecture in the HFF-STZ mice (19). Such morphological benefits which were closely linked to elevations in beta-cell proliferation (20, 34), in direct agreement with our *in vitro* observations. We also observed reductions in beta-cell apoptosis with sustained [Lys^12^Pal]Ex-4/CCK therapy that would also contribute to improved islet architecture (21). Furthermore, observed reductions in alpha-cell area and overall alpha:beta cell ratios were paralleled by decreases in pancreatic and plasma glucagon in the peptide treated mice. This observation fits well with reported glucagonostatic effects of GLP-1 receptor activation (45). However, it does contrast somewhat with the reported direct glucagonotropic actions of CCK (8), and detailed study of the overall impact of activation of GLP-1 and CCK1 receptors by [Lys^12^Pal]Ex-4/CCK in this regard is required.

Despite the obvious therapeutic attractiveness of [Lys^12^Pal]Ex-4/CCK, there are a number of significant matters that need to be considered with regards possible translation towards clinical development. Although initial fears of increased pancreatitis risk with GLP-1 mimetic therapy in humans has now been fully allayed (46), extremely high doses of CCK are employed to create experimental rodent models of pancreatic inflammation (47). However, it should be noted that the CCK1 receptor is expressed at much higher levels in pancreatic acinar cells of rodents than humans (48). Moreover, 28 days treatment with [Lys^12^Pal]Ex-4/CCK did not lead to any obvious detrimental pancreatic histopathological findings in our study. As noted above, alterations in endocrine islet morphology induced by [Lys^12^Pal]Ex-4/CCK were consistently linked to correction of the diabetes phenotype. In addition, previous studies employing sustained co-activation of GLP-1 and CCK1 receptors in rodent models reported no effect, or even reductions, in amylase and lipase secretion (25, 26). The possible impact of combined GLP-1 and CCK1 receptor activation on gastric emptying would also need to be considered (49, 50). However, we observed no obvious behavioural changes in [Lys^12^Pal]Ex-4/CCK treated mice. Finally, there is a suggestion that GLP-1 mimetics suppress CCK secretion in humans, leading to adverse gallbladder events (51). Thus, dual activation of both receptor pathways by [Lys^12^Pal]Ex-4/CCK could help to alleviate this potential problem.

In conclusion, the present study has reaffirmed clear benefits of combined GLP-1 and CCK1 receptor signalling. [Lys^12^Pal]Ex-4/CCK, created as a long-acting, dual GLP-1 and CCK1 receptor activating hybrid peptide, displayed prominent antidiabetic actions in HFF-STZ mice that were directly linked to improved islet morphology and beta-cell function. Such benefits were also coupled to induction of satiety and sustained reductions in body weight. These exciting preclinical observations necessitate the further consideration of combined GLP-1 and CCK1 receptor activation as a potential treatment option for the increasing numbers of people living with obesity and related diabetes.

## Data Availability Statement

The raw data supporting the conclusions of this article will be made available by the authors, without undue reservation.

## Ethics Statement

The animal study was reviewed and approved by the Ulster University Animal Welfare and Ethical Review Body (AWERB).

## Author Contributions

NI and PF contributed to the overall concept and experimental design, and reviewed the manuscript. AE, NT, and RL researched data, contributed to data interpretation, and edited the manuscript. NI, NT, and PF wrote the manuscript. All authors contributed to the article and approved the submitted version.

## Funding

These studies were supported by European Foundation for the Study of Diabetes (Lilly European Diabetes Research Programme) and Department for the Economy, Northern Ireland.

## Conflict of Interest

PF and NI are named on patents filed by the University of Ulster for exploitation of peptide therapeutics.

The remaining authors declare that the research was conducted in the absence of any commercial or financial relationships that could be construed as a potential conflict of interest.
